# Dataset of infrared absorption responses of leaves from five *Cannabis sativa* L. varieties treated with a *Trichoderma* spp.-based biomass suspension

**DOI:** 10.1016/j.dib.2026.112538

**Published:** 2026-02-06

**Authors:** Jorge Daniel Jara Villamayor, Fernando Lugo Pedrozo, Gabriela Romero Díaz, Mayra Lujan Vera, Camila Colmán, Nidia Balbuena, Verónica Sánchez, Edher Zacarias Herrera, Fernando Marsal, Danilo Fernández, Pablo Hernán Sotelo, Andrea Alejandra Arrúa

**Affiliations:** a Centro Multidisciplinario de Investigaciones Tecnológicas, Universidad Nacional de Asunción, Campus Universitario, San Lorenzo 111421, Paraguay; b Universidad Nacional de Misiones, Facultad de Ciencias Exactas, Químicas y Naturales, Posadas, Argentina; c Facultad de Ciencias Químicas, Universidad Nacional de Asunción, Campus Universitario, San Lorenzo 111421, Paraguay; d Facultad de Ciencias Exactas y Naturales, Universidad Nacional de Asunción, Campus Universitario, San Lorenzo 111421, Paraguay; e Brazilian Center for Research in Physics (CBPF), Rua Doutor Xavier Sigaud, 150, Urca, Rio de Janeiro, RJ 22290-180, Brazil; f Biotechnology Department, Facultad de Ciencias Quimicas, Universidad Nacional de Asunción, Campus Universitario, San Lorenzo 111421, Paraguay

**Keywords:** Hemp, Paraguay, ATR-FTIR, Trichoderma, Cannabis sativa

## Abstract

This dataset provides Attenuated Total Reflectance–Fourier Transform Infrared (ATR-FTIR) spectra of *Cannabis sativa* L. leaves from five industrial hemp varieties cultivated under field conditions in Paraguay. In total, the dataset comprises 80 ATR-FTIR spectra, corresponding to five varieties, two treatment conditions, and eight biological replicates per combination. The dataset includes raw spectral profiles from placebo-treated controls and samples treated with a *Trichoderma*-based biomass suspension, organized in individual and consolidated Excel tables. These data support varietal classification, evaluation of treatment-related spectral variation, spectral library development, and applications in quality control, chemometric analysis, and preliminary regulatory screening.

Specifications Table

**Table:** 

Subject	Biology, Chemistry
Specific subject area	*Analytical Chemistry: Spectroscopy*
Type of data	*Table, graph* Raw
Data collection	*Infrared vibrational response spectra were acquired using a spectrometer equipped with Attenuated Total Reflectance–Fourier Transform Infrared (ATR-FTIR) spectroscopy with a diamond crystal, by accumulating 128 scans at a spectral resolution of 4 cm⁻¹.*
Data source location	*Laboratory of Molecular and Elemental Analysis (MOLE), Facultad de Ciencias Exactas y Naturales, Universidad Nacional de Asunción.*
Data accessibility	Repository name: ZENODO Data identification number: 17,165,736 Direct URL to data: https://zenodo.org/records/18162715
Related research article	None

## 1 Value of the Data

• The ATR-FTIR spectra of hemp (*Cannabis sativa* L.) leaves from field-grown varieties treated with *Trichoderma*-based biomass suspensions (T) and untreated controls (C) were obtained, enabling a robust comparative analysis.
• This method is useful for varietal discrimination, evaluation of treatment effects, and development of chemometric models for quality control, authentication, and production monitoring.
• This study serves as a reference for building industrial hemp spectral libraries and supports classification studies, breeding programs, and regulatory compliance.
• The open availability of spectra facilitates reuse for machine learning training, reproducibility assessments, and benchmarking of spectral analysis algorithms.
• Supports preliminary forensic screening and comparative regulatory assessments by providing spectral profiles that may assist in distinguishing authorized hemp varieties from illicit cannabis materials.

## 2 Background

The cultivation of industrial hemp (*Cannabis sativa* L., non-psychoactive) has gained global relevance due to regulatory changes, diverse industrial applications, and its economic potential [1,2]. In Paraguay, the cultivation and research of hemp have been declared of national interest to promote its legal development and integration into productive chains, while large-scale illegal cannabis production still persists [3,4]. This context highlights the growing need for analytical tools capable of reliably supporting the differentiation between authorized hemp and illicit cannabis materials.

The genetic and chemotypic diversity of hemp varieties offers opportunities for agronomic optimization and quality control, but also poses challenges in varietal identification and product authentication [5]*.* Fourier Transform Infrared spectroscopy in attenuated total reflectance (ATR-FTIR) mode is a non-destructive technique with demonstrated capacity for spectral fingerprinting of plant materials. ATR-FTIR has been applied to varietal discrimination, evaluation of processing effects, and preliminary forensic screening and complementary regulatory assessments [[6], [7], [8]]*.* Previous studies have also reported the use of vibrational spectroscopic techniques in the analysis of pharmacological substances, with potential relevance for forensic screening [9].

This dataset provides ATR-FTIR spectra of leaves from five industrial hemp varieties cultivated under field conditions at a licensed site in Paraguay, including placebo-treated controls and samples treated with a *Trichoderma*-based biomass suspension. The standardized, openly accessible ATR-FTIR spectra are suitable for varietal classification, evaluation of treatment effects, spectral library development, and applications in quality assurance and regulatory compliance of cannabis products.

## 3 Data Description

**Fig. 1:**
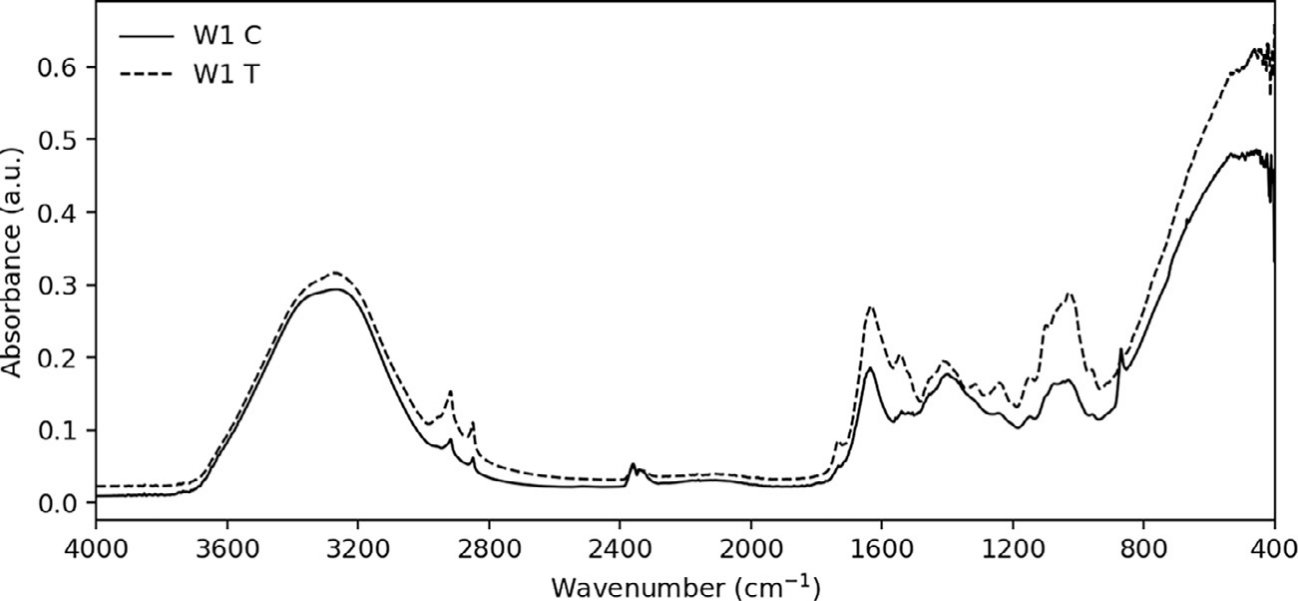
ATR-FTIR spectra of *Cannabis sativa* L. leaves from variety W1 obtained under field conditions. The placebo-treated control (W1 C) is shown as a solid black line, and the *Trichoderma*-treated sample (W1 T) as a dashed black line. Spectra are presented in the 4000–400 cm⁻¹ range.

The dataset comprises raw ATR-FTIR spectra of industrial hemp (*Cannabis sativa* L.) leaves from five field-grown varieties (coded W1–W5) cultivated under licensed agronomic conditions in Paraguay. Leaf samples were analyzed under a *Trichoderma*-based chlamydospore treatment (T) and a placebo-treated control condition (C, water application) (Fig. 1).

All spectral data are provided as raw absorbance values without baseline correction or normalization. Files are organized by variety and treatment to facilitate data handling, comparative analysis, and reuse.

The repository includes the following Microsoft Excel (.xlsx) files:

• **W1_All_spectra.xlsx – W5_All_spectra.xlsx**: ATR-FTIR spectral tables for each individual variety (W1–W5), including both *Trichoderma*-treated (T) and control (C) samples.
• **W1_T.xlsx – W5_T.xlsx**: ATR-FTIR spectral tables for each variety under *Trichoderma*-based chlamydospore treatment (T).
• **W1_C.xlsx – W5_C.xlsx**: ATR-FTIR spectral tables for each variety under placebo-treated control conditions (C).
• **W1–W5_T.xlsx**: ATR-FTIR spectral tables combining all varieties under *Trichoderma*-based chlamydospore treatment (T).
• **W1–W5_C.xlsx**: ATR-FTIR spectral tables combining all varieties under placebo-treated control conditions (C).
• **W1–W5_All_spectra_combined.xlsx:** Consolidated dataset containing all raw ATR-FTIR spectra from all varieties and both experimental conditions, enabling global comparative and chemometric analyses

Each table contains wavenumber values (cm⁻¹) in the first column and absorbance values for eight independent spectral replicates per condition in the subsequent columns.

## 4 Experimental Design, Materials and Methods

Five industrial hemp varieties (W1–W5) were cultivated under field conditions at a licensed private farm located in Aldama Cañada, Capiatá, Central Department, Paraguay, operated by WEEDZ S.A. Mature leaves were collected one month before flowering from plants treated with a *Trichoderma*-based biomass suspension (1 × 10⁶ propagules mL⁻¹ *Trichoderma asperelloides* chlamydospore formulation, T) and from placebo-treated controls (C). The treatment was applied at three defined time points: (i) at sowing, where seeds were immersed in the corresponding suspension (T or C); (ii) at transplanting, when 100 mL of the suspension was applied to the soil at the base of each plant, performed 22 days after sowing; and (iii) a final soil application of 100 mL carried out 45 days after transplanting, approximately one month before flowering. Leaf samples used for ATR-FTIR analysis were collected on the day of this final application. Subsequent leaf samplings performed after this time point are not included in the present dataset and will be reported separately. Each treatment–variety combination included leaves collected from 5 independent plants to account for biological variability. After collection, leaves were transported to the laboratory and stored at 4 °C. Due to the large number of samples, ATR-FTIR measurements were performed within a maximum of two days after sampling.

ATR-FTIR spectra were recorded with a Thermo Scientific™ Nicolet™ Summit X FTIR Spectrometer equipped with a 2 mm diamond ATR crystal, at the MOLE Laboratory, Facultad de Ciencias Exactas y Naturales, Universidad Nacional de Asunción. Spectra were collected in the 4000–400 cm⁻¹ range with a resolution of 4 cm⁻¹, averaging 128 scans per replicate for each sample. Each sample was analysed in octuplicate to ensure technical reproducibility.

A background spectrum was collected prior to each measurement series and updated every 60 min to correct for atmospheric and instrumental drift. The ATR crystal was cleaned with isopropanol and air-dried between measurements to prevent cross-contamination.

Spectral data were exported directly from the instrument software without baseline correction or normalization. All files provided in the repository contain raw ATR-FTIR absorbance spectra, organized by variety and treatment for data handling and reuse.

## Limitations

The dataset was generated under authorized conditions, as industrial hemp (*Cannabis sativa* L.) cultivation, handling, and laboratory analysis in Paraguay require prior authorization from the Paraguayan Ministry of Agriculture and Livestock [10,11]. Replication or expansion of this dataset requires strict compliance with national regulations, including the acquisition of official permits before conducting similar experimental procedures.

## Ethics Statement

This study was conducted within the framework of a doctoral thesis at the Facultad de Ciencias Químicas (FCQ–UNA) and developed at the Centro Multidisciplinario de Investigaciones Tecnológicas (CEMIT–UNA), Asunción, Paraguay. All activities were carried out under a formal resolution issued by the Comisión Interinstitucional de Cáñamo Industrial (COINCA), authorizing sample collection, transport, and laboratory analysis (Dictamen DGAJ 239/25). The work was approved by the Ethics Committee of the Centro Multidisciplinario de Investigaciones Tecnológicas (CEMIT) of the Universidad Nacional de Asunción (UNA), through Resolution No CE-00,012.

## Generative AI and AI-assisted technologies statement

During the preparation of this manuscript, the author(s) used ChatGPT to assist with translation and refinement of language. Following the use of this tool, the author(s) reviewed and edited the content as needed and take full responsibility for the content of the published article.

## Data Availability

ZenodoDataset of Infrared Absorption Responses of leaves from five Cannabis sativa L. varieties treated with a Trichoderma spp.-Based Biomass suspension (Original data).
